# Acute pancreatitis as the initial manifestation of acute myeloid leukemia with chromosome 16 rearrangements

**DOI:** 10.1007/s12185-023-03580-4

**Published:** 2023-03-25

**Authors:** Hidehito Fukushima, Ken Morita, Masako Ikemura, Mariko Tanaka, Yudai Nakai, Hiroaki Maki, Tatsunori Suzuki, Suguru Mizuno, Yousuke Nakai, Mineo Kurokawa

**Affiliations:** 1 Department of Hematology and Oncology, Graduate School of Medicine, The University of Tokyo, 7-3-1 Hongo, Bunkyo-ku, Tokyo, 113-8655 Japan; 2 Department of Pathology, Graduate School of Medicine, The University of Tokyo, Tokyo, Japan; 3 Department of Radiology, Graduate School of Medicine, The University of Tokyo, Tokyo, Japan; 4 Department of Gastroenterology, Graduate School of Medicine, The University of Tokyo, Tokyo, Japan; 5 Department of Endoscopy and Endoscopic Surgery, The University of Tokyo Hospital, Tokyo, Japan

**Keywords:** Acute pancreatitis, Acute myeloid leukemia, Chromosome 16, Sarcoma

## Abstract

Acute pancreatitis is an acute inflammatory process of the pancreas that is becoming an increasingly common clinical issue. The most frequent underlying etiologies include gallstones and chronic alcohol use, which account for more than two-thirds of cases. We recently experienced a rare case of acute myeloid leukemia (AML) presenting with recurrent acute pancreatitis, which we later discovered was caused by diffusely infiltrating extramedullary sarcoma in the pancreas. Comprehensive analysis of previous cases of AML presenting as acute pancreatitis suggested involvement of cytogenetic alterations in chromosome 16 in its pathogenesis. Further improvement in management of acute pancreatitis is needed, and clinicians should note that this occasionally fatal condition can be the initial and only manifestation of AML. In practice, prompt initiation of intensive chemotherapy is critical for treating such cases of AML-induced acute pancreatitis.

## Introduction

Acute pancreatitis is a life-threatening condition in which the pancreas becomes inflamed over a short period of time and is becoming a common clinical problem worldwide [1, 2]. Acute pancreatitis usually begins with gradual or sudden pain in the upper abdomen that sometimes extends to the back. Other symptoms can include nausea, vomiting, swollen or tender abdomen, increased heart rate, and fever [3]. Acute pancreatitis has many causes, of which gallstones and chronic alcohol use account for 70% in adults, and the remaining etiologies are broad and highly variable [4]. Recent reports have shown the complex interactions between genes and the environment in the pathogenesis of pancreatitis, and the significance of genetic variants or aberrations in the onset, severity, and outcome of pancreatic diseases has repeatedly been emphasized [5]. Practically, any factor responsible for pancreatitis can produce recurrent attacks of acute pancreatitis if not corrected, and therefore it is important to carefully evaluate the patient and address the underlying cause [1].

Among patients with acute myeloid leukemia (AML), acute pancreatitis is a rare but highly important manifestation that usually arises during the course of chemotherapies. By contrast, acute pancreatitis as the presenting complication of AML is extremely rare, and only a handful of cases have been reported to date. We recently experienced a case of AML presented with recurrent acute pancreatitis and later discovered as an extramedullary sarcoma of the pancreas, which was successfully treated with intensive chemotherapy. Besides presenting ours, in this report, we review previously reported cases of acute pancreatitis presented as a clinical manifestation of AML at diagnosis, as well as analyze the serum amylase levels from de novo AML cases that had been treated at our institute, and discuss our findings with particular emphasis on the potential involvement of cytogenetic aberrations in the chromosome 16 in its pathogenesis.

## Case description

A 35-year-old, previously healthy Japanese male presented to a neighboring hospital with periodic abdominal pain, nausea, and vomiting. Physical examination revealed epigastric tenderness without palpable abdominal mass. Laboratory tests revealed elevated levels of serum amylase (1336 U/L), lipase (109 U/L), and CRP (2.53 mg/dL), suggesting acute pancreatitis. Blood differential counts showed leukopenia with shift to the left, with the white blood cell count of 2.8 × 10^9^/L, 45% of them being segmented neutrophils. No immature myeloid cells were observed. Whole-body computed tomography (CT) scan showed an isolated 1.5 cm-diameter mass in the head of the pancreas with dilated main pancreatic duct and common bile duct upstream. The serum expression levels of CA19-9, CEA and IgG4 were within the normal range. Endoscopic ultrasound-guided fine needle aspiration (EUS-FNA) was performed, but histopathological examination failed to make definitive diagnosis as it only showed infiltration of a small number of lymphocytes into the pancreatic tissue. The etiology of the pancreatic mass remained unknown at this point.

**Fig. 1:**
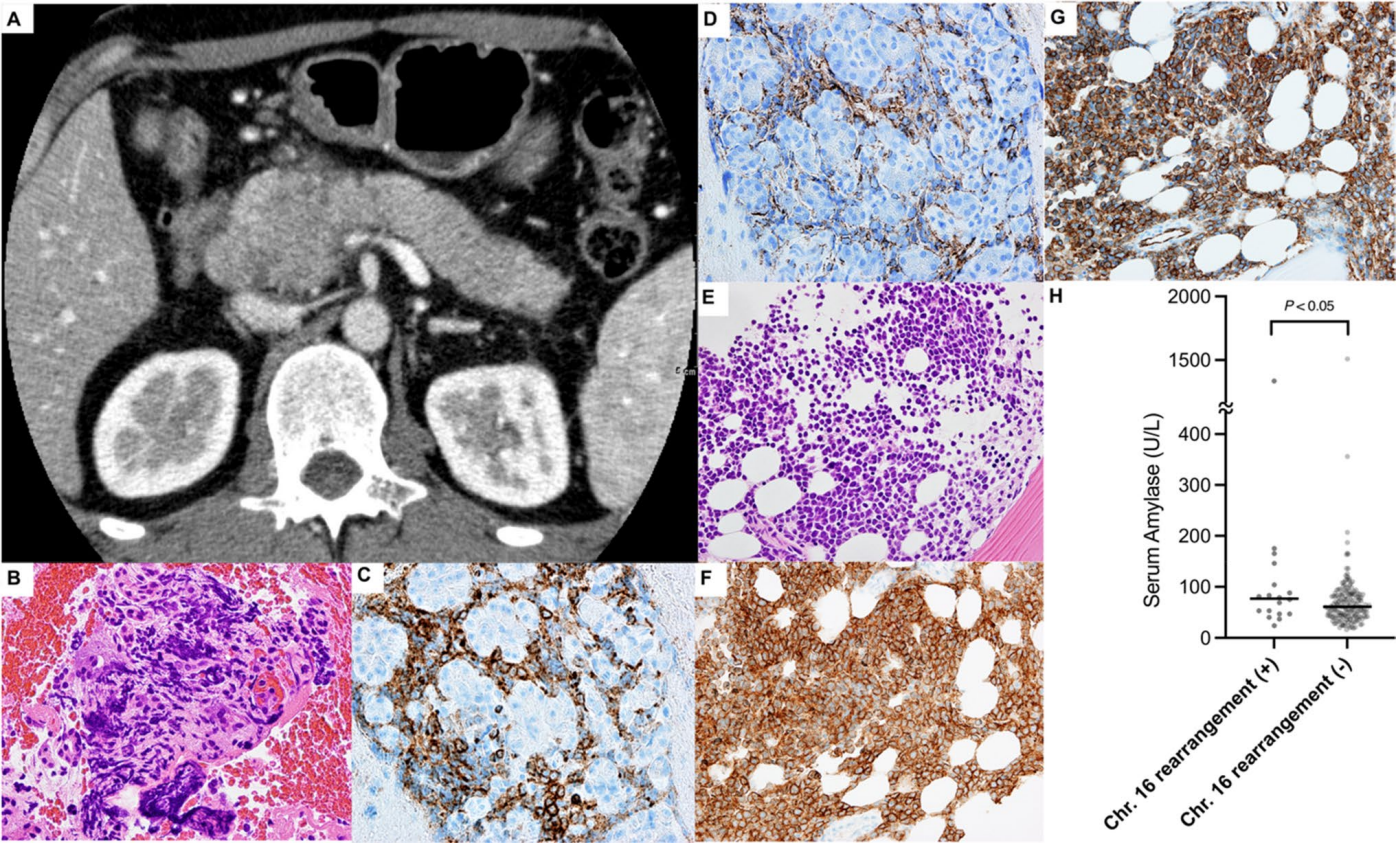
Highly Infiltrating Extramedullary Sarcoma in the Pancreatic Head. **A** Axial projection of the abdominal enhanced computed tomography scan showing an edematous pancreas due to the inhomogeneous tumor in its head. **B**–**D** Paraffin-embedded tissue sections of the pancreatic lesion showing diffuse infiltrations of intermediate-sized monomorphic myeloblasts that are positive for c-Kit and CD34, with cleaved nuclei and basophilic cytoplasm; **B** Hematoxilyn and Eosin (HE) staining; **C** immunohistochemical (IHC) staining for c-Kit; **D** IHC staining for CD34. **E**–**G** Paraffin-embedded tissue sections of the bone marrow showing diffuse proliferations of myeloblasts that are positive for c-Kit and CD34, with cleaved nuclei and basophilic cytoplasm; **E** HE staining; **F** IHC staining for c-Kit; **G** IHC staining for CD34. **H** The levels of serum amylase at diagnosis from the 196 cases of de novo AML that had been treated at our institute from January 1, 2000 to December 31, 2021. Significantly elevated levels of serum amylase at diagnosis were seen in patients with cytogenetic alterations in the chromosome 16, relative to those without it (*P* < 0.05 by two-sided student’s *t*-test; *n* = 18 for chromosome 16 rearrangement ( +) and *n* = 178 for chromosome 16 rearrangement ( −)). Among the 18 cases with chromosome 16 rearrangement, four cases (22.2%) had higher than normal amylase levels (Institute’s cut off: Serum Amylase > 132 U/L). By contrast, among the 178 cases without chromosome 16 rearrangement, only nine cases (5.1%) had such levels, indicating the higher incidence of serum amylase elevation among AML patients with cytogenetic alterations in the chromosome 16 (*P* < 0.01 by Chi square test)

Within two months of the first visit, the patient developed recurrent acute pancreatitis and was referred to our hospital for further examination. The patient complained of persistent epigastric pain radiating to the back. Physical examination revealed jaundice in the sclerae and skin and epigastric tenderness with muscular rigidity. Results of the laboratory tests showed elevated levels of serum total bilirubin (2.6 U/L), direct bilirubin (1.7 U/L), amylase (269 U/L), and lipase (665 U/L), strongly suggesting recurrent pancreatitis and obstructive jaundice. Complete blood counts showed mild thrombocytopenia (platelet counts: 13.4 × 10^4^ /μL) and normal white blood cell count of 5.1 × 10^9^ /L with the emergence of myeloid blasts (47.5%). Contrast-enhanced CT showed a 3.5-cm hypovascular mass with indistinct margins in the pancreatic head, which obstructed the common bile duct and the main pancreatic duct (Fig. 1A). The patient immediately underwent an endoscopic retrograde cholangiopancreatography (ERCP), and endoscopic drainage of the common bile duct with placement of a plastic stent was performed.

Histopathological examination of the pancreatic mass obtained under EUS-FNA revealed infiltration of the myeloblasts, which were positive for c-kit and CD34 (Fig. 1B, D). Subsequently, bone marrow aspiration and biopsy were performed, which showed markedly hypercellular bone marrow and expansion of the peroxidase-positive myeloblasts (90.8% of all nucleated cells). Myeloblasts showed high nuclear/cytoplasm ratio with indented nuclei and prominent nucleoli (Fig. 1E–G). Flow cytometric analysis revealed that these myeloblasts express CD13, CD33, CD34, CD117, MPO, CD11c, and CD56. The karyotype of the neoplasm was characterized by the translocation t(16;21) (p11.2;q22) and the addition of chromosomes 10 and 12. Accordingly, the results of the FISH analysis were negative for t(15;17) (PML/RARA), t(8;21) (AML1/ETO), or inv(16) (CBFB/MYH11). Genetic alterations for FLT3-ITD, NPM1 and DNMT3A genes were also negative in this patient. Collectively, the patient was diagnosed as AML (FAB M1) with an infiltrating extramedullary myeloid sarcoma in the pancreas. The standard induction chemotherapy was promptly initiated (cytarabine – 100 mg/m^2^/day for seven days, idarubicin – 15 mg/m^2^/day for three days). Rapid resolution of the symptoms associated with acute pancreatitis was obtained, and the contrast-enhanced CT taken on day 28 post-treatment showed markedly reduced size of the sarcoma. The patient is currently under consolidation chemotherapy, and allogenic HSCT will be performed afterwards.

## Results and discussion

Acute pancreatitis is occasionally seen among leukemia patients under active treatment, and it is most frequent among patients with acute lymphoblastic leukemia (ALL) during intensive treatment using asparaginase [5]. By contrast, symptomatic acute pancreatitis as the initial manifestation of AML at the diagnosis is extremely rare, and only 11 such cases have been reported to date (Table 1). They appear to manifest with similar clinical presentation of recurrent acute pancreatitis and are often accompanied by extramedullary myeloid sarcomas in the pancreas. Symptoms associated with acute pancreatitis responded well to the treatment for AML in most of the reported cases. Intriguingly, seven out of the eight cases (87.5%) with documented AML karyotype had genetic aberrations involving the chromosome 16, three cases (37.5%) with inv(16)(p13.1;q22)(CBFB-MYH11) and the remaining four cases (50%) with t(16;21)(p11;q22) (FUS-ERG). Of note, among the 196 cases of de novo AML that had been treated at our institute from January 1, 2000 to December 31, 2021, we found significantly elevated levels of serum amylase in the cases with chromosome 16 rearrangements, compared to those without its involvement (Fig. 1H). These findings possibly indicate subclinical infiltration of AML cells to the pancreas and subsequent local tissue inflammation in such cases with inv(16) and with t(16;21)(p11;q22). Interestingly, several reports revealed that inv(16) and t(8;21)[6, 7], but not t(16;21), are the typical chromosomal alterations in extramedullary myeloid sarcoma in general. Thus, we speculate that pancreatic sarcoma may be constructed with a unique underlying molecular mechanism to themselves, but how inv(16) and t(16;21)(p11;q22) help form pancreatic sarcomas has never been revealed due to the extreme rarity of the disease with estimated incidence of 0.01–0.05% of all AML cases[7]. So far, only a few genes might explain the bulking of myeloid blasts specifically in the pancreas [8, 9]; as Miller et al. have demonstrated in their data, the expression levels of VSIG4 are predominantly upregulated in AML patients with inv(16). VSIG4 is the immune checkpoint molecule expressed by macrophages in specific tissues, including the pancreas, and this upregulation may stimulate the tumor cells to evade tumor immunity. On the other hand, the expression levels of IGF2BP1 and SEMA3D – genes that are thought to be responsible for proliferation and activation of pancreatic adenocarcinoma – are markedly upregulated in t(16:21)(p11;q22) patients[8], suggesting that these genes may aid in the proliferation of migrating myeloblasts in a tissue-specific manner.

**Table 1:** Previously reported cases of AML presented with acute pancreatitis

References	Age/sex	AML subtype	AML karyotype	Symptoms and laboratory/imaging abnormalities associated with acute pancreatitis	Recurrent episodes (≥ 2) of acute pancreatitis	Cause of pancreatitis	Treatment for AML	Outcome
Our case	35 y/Male	M1	Complex 48, XY, + 10, + 12, t(16;21)(p11; q22)	Epigastric pain, increased serum pancreatic enzymes, diffusely infiltrating mass in the pancreatic head by CT, MRI, MRCP and ERCP	Yes	Extramedullary sarcoma of the pancreas head (EUS-FNA)	Intensive chemotherapy	Alive, 2 months after AML diagnosis
[10]	33 y/Male	N.A	46, XY, inv(16)(p13.1q22)	Epigastric pain, increased serum pancreatic enzymes, diffuse swelling of the pancreas and diffuse beaded dilatation of the main pancreatic duct by CT and MRCP	Yes	Suspected infiltration of AML cells to the pancreas	Intensive chemotherapy	Alive, 3 years after AML diagnosis
[11]	30 y/Male	M2	Complex 46, XY, del(6)(q ?), t(16;21)(p11.2;q22), add (21) (q22)	Back pain, increased serum pancreatic enzymes, swelling of the pancreatic body by CT	Yes	Documented infiltration of AML cells to the pancreas (EUS-FNA)	Intensive chemotherapy, HSCT	Dead, 2 years and 9 months after AML diagnosis
[12]	37 y/Female	N.A	Complex involving t(16;21)(p11;q22)	Epigastric pain, increased serum pancreatic enzymes, diffuse swelling of the pancreas and a mass in the pancreatic head by CT	Yes	Suspected extramedullary sarcoma of the pancreatic head	Intensive chemotherapy, HSCT	Dead, 6 years and 4 months after AML diagnosis
[13]	19 y/Female	N.A	inv (16)(p13.1q22)	Epigastric pain, nausea and vomiting, increased serum pancreatic enzymes, and a mass in the pancreatic head by CT, PET-CT and EUS	No	Extramedullary sarcoma of the pancreas head (EUS-FNA)	Intensive chemotherapy, HSCT	Alive, 2 years after AML diagnosis
[14]	75 y/Female	M4eo	inv (16)	Epigastric pain, increased serum pancreatic enzymes, and a diffusely infiltrating mass in the pancreatic head by MRI	No	Suspected extramedullary sarcoma of the pancreatic head and diffusely infiltrating AML cells	Intensive chemotherapy	Dead, 7 months after attaining CR
[15]	39 y/Male	N.A	trisomy 8, t(16;21)	Abdominal pain radiating to the back, diffusely abnormal pancreas, and enlarged peripancreatic lymph nodes by EUS	Yes	Suspected infiltration of AML cells to the pancreas	Intensive chemotherapy, HSCT	Dead, 11 months after AML diagnosis
[16]	47 y/Male	M5A	t(8;17) and t(17;17)	Epigastric pain, increased serum pancreatic enzymes, and a mass in the pancreatic head with edematous pancreas from head to tail by CT and EUS	Yes	Diffusely infiltrating Extramedullary sarcoma of the pancreas head (laparotomy)	Intensive chemotherapy	Alive at time of report
[17]	19 y/Male	N.A	N.A	Epigastric pain, increased serum pancreatic enzymes, and a diffusely infiltrating mass in the pancreatic head by CT, MRI and PET-CT	No	Documented infiltration of AML cells to the pancreas (EUS-FNA)	Intensive chemotherapy	Alive at time of report
[18]	36 y/Male	N.A	N.A	Epigastric pain, increased serum pancreatic enzymes, and a diffusely infiltrating mass in the pancreatic tail by CT, EUS and PET-CT	Yes	Documented infiltration of AML cells to the pancreas (percutaneous FNA)	Intensive chemotherapy	Alive, 1 year and 7 months after AML diagnosis
[19]	19 y/Female	N.A	N.A	Epigastric pain, increased serum pancreatic enzymes, and a mass in the pancreatic head by MRI	Yes	Extramedullary sarcoma of the pancreas head (laparotomy)	Intensive chemotherapy, HSCT	Dead, 1 year and 8 months after AML diagnosis

Eleven cases of the previously reported AML presented with acute pancreatitis are listed

AML subtype is shown for each of the cases according to the French-American-British (FAB) classification

*N.A.* not available, *HSCT* hematopoietic stem cell transplant, *PET-CT* positron emission topography and computed tomography, *EUS* endoscopic ultrasound, *FNA* fine needle aspiration biopsy, *CR* complete remission

These observations collectively suggest the involvement of chromosome 16 aberrations in the pathogenesis of acute pancreatitis among AML patients. Whole genome sequencing was not routinely performed at the time of diagnosis in this case series, and we are not sure if there is a common undetected genetic alteration in these cases. We consider this to be one of the limitations of this study and wish to make further research. Hopefully, our findings stimulate future biological investigations to examine our hypothesis experimentally.

In summary, we experienced a rare case of AML presented with acute pancreatitis induced by an extramedullary myeloid sarcoma in the pancreatic head. Our comprehensive review of the previous reports and analysis of the serum amylase levels from the 196 de novo AML patients who had been treated at our institute indicate higher incidence of acute pancreatitis among AML patients with cytogenetic abnormalities in the chromosome 16 besides suggesting its involvement in the pathogenesis of acute pancreatitis in AML patients. Thus, although rare, active search for AML should be considered in patients with acute pancreatitis, especially in cases with recurrent episodes of acute pancreatitis of unknown etiology.

## Data Availability

Relevant clinical data is available upon request.
